# Failure of Cone-Beam Computed Tomography in Detection of Fiber Post Perforation

**DOI:** 10.22037/iej.v12i3.17690

**Published:** 2017

**Authors:** Mahta Fazlyab, Saeed Asgary

**Affiliations:** a * Department of Endodontics, Dental Branch, Islamic Azad University, Tehran, Iran; *; b *Iranian Center for Endodontic Research, Research Institute of Dental Sciences, Dental School, Shahid Beheshti University of Medical Sciences, Tehran, Iran*

**Keywords:** Calcium-Enriched Mixture, Cone-Beam Computed Tomography, Diagnosis, Post Space Preparation, Root Perforation

## Abstract

Detection of iatrogenic root perforation during post-space preparation especially in labiolingual plane can be challenging due to the two-dimensional nature of conventional radiography; this can be even more challenging if the cemented post is radiolucent. Cone-beam computed tomography (CBCT) scans were shown to be a valuable diagnostic aid in diagnosis of such cases. However, in this case, the application of CBCT did not help in diagnosis of a labial fiber post perforation in a maxillary central incisor which was finally detected through exploratory surgery.

## Introduction

Root perforation refers to creation of an artificial and unwanted communication between the root canal system and the periodontal ligament (PDL); at the site of root perforation, an inflammatory reaction in PDL occurs and leads to the formation of a primary endodontic lesion [1]. Perforation along the root can occur as a result of root resorption, iatrogenic damages during root canal therapy (RCT) and finally during post-space preparation [2]. Iatrogenic root perforations frequently caused by inappropriate post space preparation are amongst the most common types of root perforations [3] occurring approximately between 2 to 12% of the endodontically treated teeth [4]. 

Accidental and unnoticed lateral root perforations during post-space preparation, show lateral bone defects on radiographies and cause formation of a sinus tract or periodontal pocket [5]. Occasionally, radiographic diagnosis of this defect in a patient with non-acute clinical symptoms is challenging as periapical radiographies show previous endodontic treatment, presence of a post and a crown which is restored without evidence of a root perforation caused during the post insertion [5]. Radiographic detection of the labial or lingual root surface perforation is even more challenging, due to superimposition of the perforation on the root [2]. In such cases, tube shift technique with at least two different horizontal angles can be helpful in identification of a misdirected post. The greatest limitation of conventional radiography, which is inability to represent the three-dimensional (3-D) anatomy of teeth and their related structures, has been overcome by introduction of cone-beam computed tomography (CBCT) [6]. However, if the cemented post is radiolucent, its detection seems almost impossible not radiographically nor in CBCT.

Happening for any reason, this unwanted communication leads to destruction of the adjacent periodontal tissues [7]. The survival prognosis of perforated tooth depends foremost on prevention or control of bacterial (re)infection and epithelial migration at the perforation site [5]. Use of a biocompatible repair material to provide the best possible seal against penetration of bacteria and the ability to promote regeneration of cementum and periodontal ligament will limit periodontal inflammation [8, 9]. 

**Figure 1.**
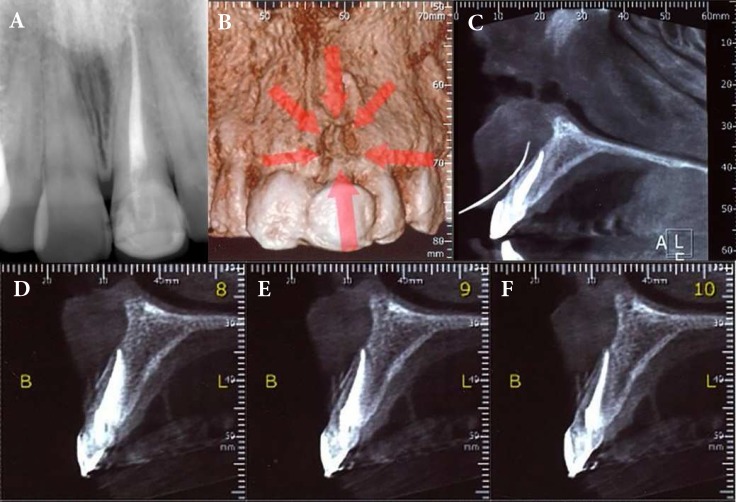
*A)* Periapical radiography shows flawless RCT; *B)* Three-dimensional reconstruction provided by the CBCT software showing a bony lesion; *C)* Deviation of the tracing gutta-percha cone to the labial soft tissues; *D-F)* Normal CBCT findings

Calcium-enriched mixture (CEM) cement is a tooth-colored cement with clinical applications similar to MTA. It offers proper sealing ability, biocompatibility, antimicrobial properties, hard tissue induction properties and shorter setting time, greater flowability and lower film thickness than MTA [10-13].The present report reviews the challenging diagnosis and surgical treatment (using CEM cement) of a radiolucent post-space root perforation on labial surface that had developed a sinus tract. In this report the inability of CBCT and efficacy of exploratory surgery in detection of the lesion, is highlighted.

## Case Report

A young women in her early twenties was referred by a dentist, complaining of localized discomfort in the anterior maxillary vestibule subsequent to endodontic treatment and restoration of the left maxillary central incisor. Her medical history was noncontributory. Upon clinical assessment the buccal gingiva of the maxillary left central incisor had a sinus tract in the mid root area which according to the patient was occasionally formed after RCT. In the referral letter, the dentist had mentioned RCT was indicated after detection of pulp necrosis of the incisor which had a class three cavity. The tooth was not sensitive to percussion. A parallel periapical radiography was taken that indicated perfect RCT and restoration (Figure 1A). Due to root fracture being likely, cone-beam computed tomography (CBCT) was indicated. At the first glance, normalcy was seen in different CBCT slices. However, a fenestration like lesion was detected in the three-dimensional reconstruction provided by the CBCT software (Figure 1B, red arrows). The sinus tract was traced with a gutta-percha cone and CBCT was taken which did not show apical origin of the lesion. In other words, deviation of the tracing gutta-percha cone to the labial soft tissues in CBCT image (Figure 1C) intensified the diagnosis.

As the CBCT findings were not suggestive of the lesion origin (Figure 1D to E), exploratory surgical flap was indicated. After buccal infiltration of 2% lidocaine containing 1:80000 epinephrine (Darupakhsh, Tehran, Iran), a submarginal Ochsenbein-Luebke flap was resected and reflected. 

**Figure 2:**
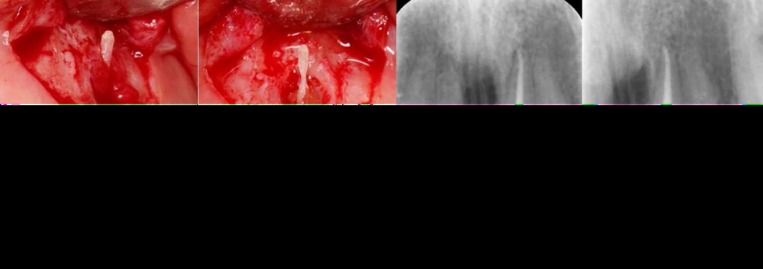
*A* *and* *B)* A flap was reflected and showed a fiber-reinforced lucent prefabricated post that had perforated the root and bone just under the gingival soft tissues; *C)* Post-surgical periapical radiographic image of the tooth; Note the CEM filling in the mid-root area bellow the cervical margin (arrow head); *D)* The one year follow-up radiography was also indicative of a normal tooth

Interestingly, after curettage of the granular tissue surrounding the fenestration, a fiber-reinforced lucent prefabricated post became visible that had perforated the root and bone just under the gingival soft tissues (Figures 2A and B). That segment of the post which was accessible outside the root was resected and after preparation of a small cavity inside the root, the area was sealed with CEM cement (BioniqueDent, Tehran, Iran), (Figure 2C). The flap was replaced and sutured. After giving the post-treatment instructions the patient was dismissed. After 5 days, patient was totally asymptomatic and the sutures were removed. After two weeks the sinus tract had disappeared and the tooth was functional. The one year follow-up radiography was also indicative of a functional tooth with no pathology (Figure 2D).

## Discussion

The present report reviewed successful surgical management of a mid-root perforation during post space preparation in an upper left central incisor that was left undetected by the dentist. The post lucency prevented its emergence in radiographies and contrary to our perception, CBCT was not much helpful in detection of the lesion origin. Thus, exploratory surgery was indicated which uncovered the cause of patient’s problem; labial mid-root perforation during post space preparation where the radiolucent fiber post was cemented. The perforation site was prepared/sealed using CEM cement. After one year, the tooth was symptomless and functional.

Root perforation often happens iatrogenically as a result of misaligned application of burs during endodontic access preparation and search for root canal orifices, and during efforts to negotiate calcified and curved canals [2, 4]. Inappropriate post space preparation for permanent restoration is yet another common iatrogenic cause of root perforation [4]. Kvinnsland *et al.* observed that more than half of the perforations in their cases of root perforation occurred during post-preparation procedures [3]. 

In some cases of endodontic practice, conventional intraoral radiography does not provide adequate information on pathologic conditions and positional relationships. Three-dimensional images have become possible with introduction of CBCT technology to dentistry. The imaged area can be arbitrarily sliced and observed from three different directions [6]. Although the presence and position of a fenestration and associated bone defect were confirmed preoperatively and in three-dimensionally reconstructed CBCT image, examination with CBCT could not reveal clear evidence of a post perforation through the labial root surface of tooth 21 which could remain undetected if exploratory flap had not been indicated. In some cases when the diagnosis is doubtful and the evidence for root fracture or perforation is tenuous, it may be necessary to perform an exploratory flap [14]. In other words, it may be very reasonable to consider surgical exposure unless the nonsurgical evidence clearly indicates the presence of a fracture/perforation whose extent or position mitigates against any possibility of treatment [15]. Management of complicated endodontically treated teeth requires special consideration, particularly if the clinician is unable to elicit the proper past history, such as where, when, and how the tooth in question was treated. In such cases an approach more than just opening the pulp chamber and root canal(s) is required for a correct diagnosis [16]. Thus exploratory endodontic surgery may become necessary to help determine prognosis and plan treatment by better evaluation of the damage through surgical exposure and direct visual examination of the area [15, 16]. However, to the best of our knowledge almost all reports of successful exploratory surgeries explained its usefulness in detection of root fractures [15, 16], resorption [17] and cemental tear [18]. Thus, this is the only report that represented indication of exploratory flap in detection of post space root perforation. In the reported case, the lesion origin was clear after raising an exploratory flap due to post material being radiolucent. However, the operator was ensured of the presence of pathogenesis in the affected area. In addition, the CBCT images made all the procedure justifiable for patient. 

Many important factors that are all related to infection control at the perforation site, influence the survival prognosis of the tooth [19, 20]: the size of the perforation, the time elapse between creation of the perforation and its repair, and the site of perforation in relation to the level of crestal bone and epithelial attachment [21, 22]. As the most critical factor determining outcome of treatment, time indicates the chance of infection of the wound site [19, 20] while defect size relates to amount of trauma to the adjacent tissues and difficulty in creating seal in the affected area [9]. Perforations occurring close to the crestal zone may be complicated by formation of a periodontal pocket due to epithelial migration that forms a connection between the perforation defect and the oral cavity [20]. Down-growth of gingival epithelium to the most apical margin of perforation site can emerge, especially when accidental perforations occur in the crestal area [4, 20]. Persistence of bacterial infection originating from the root canal and/or the periodontal tissues, prevents healing and brings about inflammatory sequels where exposure of the supporting tissues is inflicted. Thus, painful conditions, suppurations resulting in tender teeth, abscesses and sinus tract including bone resorptive processes may follow [3, 20]. According to Patel *et al.* [23] perforation of the root is usually followed by the development of a sinus tract. In the presented case, the perforation was located in the mid-root area far from the crestal bone and luckily did not cause pocketing to the perforation site. Consequently, sealing of the defect was successful in controlling the intra-radicular infection.

CEM cement has been recommended as an appropriate endodontic biomaterial for repair of lateral root perforations [8, 9, 11]. Comparison of the results of this study with the results of reports on root perforations repaired with CEM cement shows a marked improvement in the prognosis of these teeth [7, 8, 24, 25]. In the case reported here, healing was achieved following repair of the lesion associated with a sinus tract and a bone defect with CEM cement.

## Conclusion

In conclusion, the present case report showed that a surgical approach may be effective for treating a combined endodontic-periodontal lesion originating from a root perforation in the middle third of a maxillary central incisor with a cement lucent post, which was treated with a surgical approach using CEM cement. The presented case demonstrates that CBCT imaging technology is not always useful in detection of lesions that are not clinically and radiographically diagnosable. Endodontic surgical flaps for diagnostic purposes and management of complex endodontic problems cannot be overlooked.
